# Psychosocial Characteristics and Experiences in Patients with Multiple Endocrine Neoplasia Type 2 (MEN2) and Medullary Thyroid Carcinoma (MTC)

**DOI:** 10.3390/children9060774

**Published:** 2022-05-25

**Authors:** Robin Lockridge, Sima Bedoya, Taryn Allen, Brigitte C. Widemann, Srivandana Akshintala, John Glod, Lori Wiener

**Affiliations:** 1 Clinical Research Directorate (CRD), Frederick National Library for Cancer Research, Frederick, MD 21701, USA; robin.lockridge@nih.gov; 2 Pediatric Oncology Branch, National Cancer Institute, National Institutes of Health, Bethesda, MD 20892, USA; sima.bedoya@nih.gov (S.B.); taryn.allen@nih.gov (T.A.); widemanb@mail.nih.gov (B.C.W.); srivandana.akshintala@nih.gov (S.A.); john.glod@nih.gov (J.G.)

**Keywords:** medullary thyroid carcinoma, psychosocial, pediatrics, young adults

## Abstract

Multiple Endocrine Neoplasia type 2 (MEN2) is a genetic cancer syndrome for which there are limited data pertaining to the quality of life and psychosocial experiences of persons affected. Medullary thyroid carcinoma (MTC) is a rare disease of the thyroid gland often associated with MEN2. MTC often progresses slowly and may present with a myriad of physical symptoms including hair loss, sleep disturbance, fatigue, weight changes, heart palpitations, and constipation or diarrhea. Like other cancers or rare, inheritable illnesses, patients with MEN2 and MTC may be at risk for psychosocial stressors. The current, cross-sectional study administered a structured psychosocial interview and The Distress Thermometer/Problem Checklist to 63 patients with MEN2 and MTC and their caregivers. Despite reports of overall good health, 46% of adults and 44% of youth reported that pain interferes with their daily life; 53% of adults and 59% of youth reported that pain interferes with their mood. Pediatric patients frequently reported experiencing attention challenges (50%) and difficulty concentrating (65%). Parents reported that mood shifts and becoming upset easily were the most prevalent concerns for their children. The most frequent need for services included education about MTC, treatment and research participation, and the opportunity to meet others with MTC.

## 1. Introduction

Multiple Endocrine Neoplasia (MEN) 2A and 2B are rare cancer predisposition syndromes resulting from germline mutations of the Rearranged during Transfection (RET) oncogene [1]. In children and young adults, MEN 2A and 2B is frequently associated with Medullary thyroid carcinoma (MTC), a rare malignancy derived from neural crest-derived parafollicular C cells of the thyroid gland. It accounts for 3–10% of all thyroid carcinomas [2,3]. Sporadic MTC (in the absence of germline RET alteration) is typically seen in middle-aged adults [4,5,6,7]. MEN 2A is characterized by hereditary MTC in almost all affected individuals. Additionally, around half of affected individuals also develop pheochromocytoma, and approximately 15% may also develop hyperparathyroidism. Patients can present as early as five years of age, but typically present between the ages of 15–20, and age of disease onset and symptom phenotype is influenced by the type of RET mutation [8,9]. MEN 2B is less common than MEN 2A, but is a clinically more aggressive form, and it also presents in the earlier years of life. MTC develops in virtually all patients with MEN 2B and is the leading cause of death in these patients. Patients with MEN 2B may also have gastrointestinal (GI) dysmotility and abnormal dilation of the GI tract such as megacolon or megaesophagus. Skeletal deformities such as slipped capital femoral epiphysis (SCFE), scoliosis, pectus, and foot abnormalities may also be associated with MEN 2B [4,5,10,11,12]. 

MTC is the most common cause of death in patients with MEN 2A and MEN 2B, as there are limited treatment options for advanced or metastatic disease and the tumor is relatively unresponsive to conventional doses of radiation therapy and to standard chemotherapeutic regimens [2,13,14,15,16,17,18,19,20,21]. Tyrosine kinase inhibitors that block RET activity are effective in treating patients with MTC, but resistant disease can develop [22]. Thyroidectomy performed at an early age when the tumor is confined to the thyroid gland is the only curative treatment for patients with MTC. In patients with known family history, early recognition through genetic screening and detection of one of the characteristic mutations followed by prophylactic thyroidectomy has become the standard of care [23,24,25]. However, in many instances, particularly in patients with MEN2B, there is no known family history, and patients are diagnosed with more advanced disease that cannot be cured by surgery alone [26].

MTC is often described as having a chronic and indolent disease process because it progresses slowly, over years or decades, with or without symptoms. Like other cancers, over time MTC has the potential to significantly impact the physical as well as emotional, social, and financial well-being of diagnosed individuals and their loved ones. Patients may suffer from symptoms related to iatrogenic hypothyroidism such as hair loss, sleep disturbance, fatigue, weight changes, heart palpitations, temperature sensitivity, and constipation or diarrhea. Psychological symptoms may include inability to concentrate, depression, or anxiety. As MTC in association with MEN 2 is an inheritable disease, unique psychosocial stresses associated with this disease have been noted as important to investigate [27,28]. 

Studies have examined the psychosocial aspects associated with genetic testing [27,29] and the impact of being at risk of MTC on patients’ quality of life [28,30]. Additionally, psychological distress, coping, and quality of life have been assessed in patients with MEN2 [28,31,32,33]. However, few studies have explored whether there are unique parental concerns and family stresses associated with MTC compared to those for parents of children with other pediatric cancers. Similarly, there are no data to examine whether the stresses for youth living with MTC and their family members change over time, or if concerns differ across the lifespan. This is an area of particular concern given the potential impact on fertility and risk of passing on MEN in future children.

As part of a larger NIH IRB approved study designed to develop a better understanding of the biology and natural history of MEN 2 with or without MTC in children and young adults, we aimed to learn about the psychosocial experiences of this patient cohort. 

## 2. Materials and Methods

### 2.1. Participants

Pediatric and adult patients with histologically or cytologically confirmed MTC, or those with known MEN2 syndrome (with or without MTC) who were able to travel to the NIH and undergo evaluations, were eligible for the natural history study. Exclusion criteria included not being able to return for follow-up visits, obtain required follow-up studies, or sign a written informed consent document.

All patients enrolled in the natural history study were invited to participate in the collection of psychosocial measures. A psychosocial provider (L.W., S.B.) met with each patient during their visit, during which time the measures were completed. Pediatric and young adult patients (≥12 years) completed self-report measures and parents/caregivers (referred to as parents from here on) of children or young adults of all ages completed proxy measures. However, for this sample, parent responses were only included for patients under 18 years of age. Notably, responses were collected and included from one parent only. All participants provided consent or assent, when applicable, or consent was obtained from their parent or legal guardian. 

### 2.2. Measures

Structured Psychosocial Assessment Interview: As no specific standardized instrument assessing how MTC impacts quality of life was available, a structured self-report assessment was designed for the overall study. A version of the structured psychosocial assessment was developed for the NIH Gastrointestinal Stromal Tumor (GIST) Clinic in order to identify specific psychosocial areas of concern and self-identified patient-related needs [34]. The assessment was adapted for the MTC cohort after conducting a literature review related to MTC and psychosocial functioning. It contains items covering demographic factors, family stressors, general health, psychosocial concerns, psychiatric history, self-identified needs, expectations regarding disease outcome and positive events that might have occurred since diagnosis, and interest in a range of possible psychosocial services [34]. To enhance the face validity of the data, the questions were checked by medical and nursing staff experienced in the care of persons living with MTC. Three versions of the assessment were created: one for adult patients (age ≥ 18 years), one for parents of children with MTC to complete about their child, and a third, shorter assessment for adolescent patients (ages 12–17 years). 

Distress Thermometer: The Distress Thermometer (NCCN, 2008) is a brief screening tool endorsed by the National Comprehensive Cancer Network (NCCN) to assess for distress in adult cancer patients. The Distress Thermometer (DT) is a visual-analog scale similar to those used to assess pain. The scale ranges from 0 (No Distress) to 10 (High Distress) and includes a “problem list” where patients can identify the specific reasons for their distress. The DT has been widely validated in adult (≥18 years) cancer patients, recognized as a good alternative to many of the longer measures commonly used to screen for distress in cancer patients [35], and has been adapted and validated in pediatric patients with cancer and other serious conditions [36,37,38]. The DT was further adapted for this protocol to include some of the specific issues thought to potentially cause distress in patients living with MTC, including body image, pain, weight gain or loss, and gastrointestinal concerns. Two versions of the DT have been developed for this protocol, one for parents of children to complete about their child, and a second for adolescent and young adult patients. As the questions contained in both the psychosocial assessment and on the DT problem list are not developmentally appropriate for children under the age of 12, data for children younger than 12 years were obtained though parent report only. 

### 2.3. Statistical Analysis

Data were analyzed using IMB SPSS 27 statistical software. Unless otherwise indicated, when items contained missing responses, the valid percent was reported. 

Qualitative analyses were conducted on open-ended, free-text narrative responses. These responses were analyzed by two authors (R.L., S.B.) to identify common themes. The authors met to refine themes and develop codes for analysis (Macqueen et al., 1998). Free-text responses were then coded in parallel (R.L., S.B.) with differences resolved through consensus discussion. Responses that were judged to fall under one or more thematic categories were coded under all applicable themes. 

## 3. Results

### 3.1. Characteristics of the Total Sample

#### 3.1.1. Demographic Characteristics

Sixty-three patients participated in this study; 77.8% (*n* = 49) were diagnosed with MEN2B and 22.2% (*n* = 14) were diagnosed with MEN2A. Additionally, 82.5% (*n* = 52) had also been diagnosed with MTC at the time of this study. As shown in Table 1, the total patient sample was largely pediatric (73%) and predominantly white (71.4%). The sample was comprised of 31 males and 32 females. Over half of adult participants had graduated high school or received an equivalent degree (28.6%), completed some college or vocational school (21.4%), or graduated college or vocational school (28.6%). Parents of pediatric patients (*n* = 46) were predominantly mothers (71.1%), married (80%), and highly educated, with 28.9% having graduated from college or vocational school and 20% having completed a professional or graduate degree.

#### 3.1.2. Clinical and Mental Health Characteristics

Parents and adult and pediatric patients were asked to indicate whether they or their child had experienced any mood, psychological, or social difficulties over the past month. As shown in Table 2, the most frequently reported areas of concern indicated by adult patients (*n* = 15) with MEN2 or MTC were a tendency to “cry or become upset easily” (40%), “feeling sad or depressed” (33.3%), “difficulty concentrating” (33.3%), and “anxiety or panic attacks” (26.7%). As shown in Table 3, 3 of 17 adults reported currently being under the care of a mental health provider for therapeutic or prescription-based treatment. However, despite reporting very few symptoms on the psychosocial assessment, of adult patients that provided responses (*n* = 15), over half (66.6%) indicated moderate to severe distress over the past month on the DT scale. The average overall distress rating was 5.0 (SD: 2.8, Range 0–10). Of those reporting moderate to severe distress (*n* =10), the predominant areas of concern were “feeling worried or anxious”(80%), “pain” (70%), and fatigue or lack of energy (80%). 

Psychosocial symptoms were reported more frequently amongst pediatric patients and their parents. “Difficulty concentrating” (65.4%) and “attention challenges” (50.0%) were among the most prevalent symptoms identified by pediatric participants. In contrast, as shown in Table 4, parents across pediatric age groups reported that “mood shifts” and a tendency to “cry or become upset easily” were the most frequent challenges for their children. Despite the increase in symptom reporting, the pediatric population remained similar to adults in that a much smaller proportion of those reporting symptoms were receiving any form of therapeutic treatment for their psychosocial concerns at the time of the study. Twenty-six adolescent patients (ages 12–17) provided responses to the DT scale; the average distress rating was 4.27 (SD: 2.6, Range: 0–10). Half reported scores within the moderate to severe range. Among those patients (*n* = 13), “feeling worried or anxious” (76.9%) and “schoolwork” (69.2%) were the most frequently reported sources of distress. Doctor/hospital visits, pain, and difficulty concentrating were also reported by just over half of the pediatric sample (53.8%). Parents of adolescent patients with MTC were also asked to indicate their child’s distress over the past month and they reported an average distress rating of 4.48 (SD:2.5; Range 0–10). Sixty-one percent endorsed scores within the moderate to severe range. Within this subgroup of parents, “worry and anxiety” (63.0%), “fatigue” (55.6%), “frequency of doctor and hospital visits” (59.3%), and “parental stress” (59.3%) were among the most distressing experiences for their child. 

### 3.2. Perceptions of Physical Health and Pain

**Figure 1:**
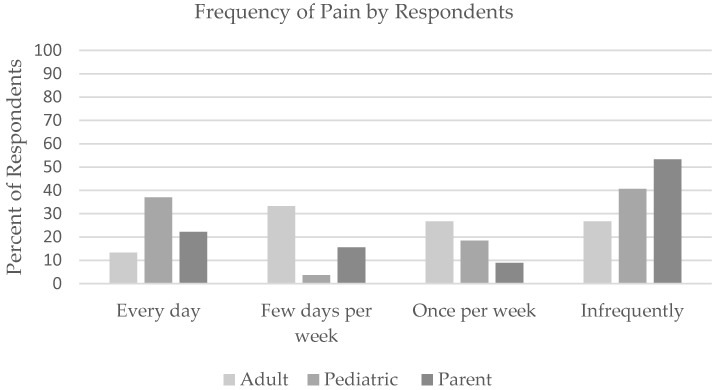
Ratings of pain frequency as reported by adult and pediatric self-report population, and parents of pediatric MTC patients of all ages. Pediatric self-report *n* = 27; parent report *n* = 45 Adult self-report *n* = 15.

Most adults living with MTC reported that their overall physical health was in ‘good’ (40%) ‘very good’ (26.7%), or ‘excellent’ (6.7%) condition. Twenty-three percent of adults that provided responses (*n* = 15) reported their overall health was ‘fair.’ Over half of adults reported experiencing pain at least once per week (Figure 1). Forty-six percent reported that pain interfered with their daily lives and fifty-three percent reported that pain interfered with their mood.

The majority of pediatric participants living with MTC reported to be in ‘good’ (37%), ‘very good’ (33.3%), or ‘excellent’ (14.8%) physical health, while 14.8% reported their physical health as ‘fair.’ Although 40.7 percent of the pediatric cohort reported experiencing pain ‘infrequently,’ a similar proportion (37%) reported experiencing pain everyday (Figure 1). Forty-four percent of youth reported that pain interferes with their daily life, and 59.3 percent reported that pain interfered with their mood. Fifty-three percent of parents reported their child experienced pain ‘infrequently’ and that pain did not interfere with their lives. However, 46.7% of parents reported that pain interfered with their child’s life, and just over half (51.1%) reported that pain did interfere with their child’s mood. 

### 3.3. Qualitative Data and Interest in Supportive Resources

In an open-ended question, patients with MTC and their parents were asked to identify the three most difficult parts of living with MTC. Forty-four patients (adult and pediatric) and forty-four parents provided at least one response. Three consistent themes emerged: (1) disease-related experiences and challenges, (2) internalized experiences of living with MTC, and (3) external impact of living with MTC, under which nine codes were developed. Example responses can be found in Table 5. 

*Diagnosis specific concerns* Patients with MTC and their parents described the learning of and presence of the illness itself, rarity of the disease, and limited treatment options as particular challenges related to their diagnosis.

*Treatment Related Impact* One of the most prevalent codes was related to treatment-related experiences. This code highlighted patient experiences and difficulties with numerous hospital and doctor visits, surgeries, medication management, and medical tests.

*Symptom Impact* Physical symptoms and the impact of physical symptoms were recurring concerns identified by patients. These frequently included pain, gastrointestinal (GI) symptoms, and sleep disturbance. 

*Physical Limitations* Strength, mobility, and difficulty engaging in physical activities were notable areas of concern for participants. Participants often remarked about feelings of weakness and difficulty keeping pace with their same-aged peers. 

*Mental Health Impact* Participants described feelings of sadness, anxiety, stress, and general decline in social-emotional wellbeing as a result of diagnosis, treatment, or ongoing management. 

*Coping with Uncertainty* Patients and parents often referred to their discomfort surrounding the uncertainty of the future and how the presence of MTC and potential for disease progression would continue to impact their lives and future plans. Participants also described feelings of uncertainty as they waited for results from diagnostic scans. Parents reported uncertainty or feelings of guilt surrounding their genetic mutation and specifically, the impact on their child’s health and future. 

*Being Different* Participants noted the difficulty of having to cope with feeling and appearing different than their peers. Differences were attributed to both physical attributes (e.g., mucosal neuromas, bumps on the lips or tongue) as well as the inherent difference of living with MTC, a rare disease, that is not present in the lives of their peer groups. 

*Social Impact* The impact of living with MTC on social interactions was identified as an area of difficulty. Participants described embarrassment surrounding the presence of symptoms, including challenges participating in social activities due to symptom management or medical care appointments. Participants also reported concerns that others would feel sorry for them. 

*Family Impact* Participants described several areas within their families that have been impacted by their MTC diagnosis. Worries about parental and sibling stress, finances, and equally dividing attention amongst affected and unaffected siblings were described. 

*School/Work Impact* Difficulty attending or completing and managing school or work assignments alongside multiple hospital visits or doctor appointments were reported as consistent hardships. 

Amongst parents of children with MTC, the most frequently endorsed needs for services were education about MTC, MEN 2, treatment options or current research (92.7%), the opportunity to meet other patients with MTC or MEN 2 (76.9%), and a support group for themselves or other family members (60.0%). Pediatric and adult patients also reported interest in opportunities to meet others with MTC or MEN 2 (Pediatric: 76.0%, Adult: 53.3%) and additional education about MTC, MEN 2, and treatment options (Pediatric: 57.7%, Adult: 73.3%). 

## 4. Discussion

Although the current sample largely reported good to excellent physical health, several interesting results emerged surrounding the utility of supportive services, the impact of pain, and patients’ own description of their unique daily challenges. Despite reporting moderate to severe distress in the last month, adult participants generally reported very few mental health symptoms, which was consistent with their current utilization of mental health treatment services. Our results are in contrast with data from adults living with MEN2, which found frequent symptoms of anxiety and depression and indicated that psychological distress is a chronic symptom for adults with MEN2 and is likely due to a number of MEN2-related factors [31]. It is possible that our sample has developed effective coping strategies over time and that the stress they are reporting is intermittent and not atypical. Conversely, access to mental health support due to financial or other resource barriers may be limited. These results are only a snapshot of the patient experience at one timepoint. In order to evaluate whether effective coping strategies among MTC patients improves with age, it will be important to continue to collect and analyze longitudinal data.

Pediatric patients reported more functional symptoms (inattention and difficulty concentrating) while their parents noticed more mood concerns (e.g., crying, mood shifts). It is well-known that anxiety and worry can present as inattention or difficulty concentrating, which suggests that the pediatric group’s symptom endorsements are consistent with some of their most frequent sources of stress in the past month. Despite the apparent prevalence of these challenges in their daily lives, it is quite notable that very few participants had received any current mental health treatment. Pediatric patients did report receiving special education support at a proportion that is higher than the current United States (U.S.) national average [39]; however, the extent of supportive services (e.g., pull-out services, reduced assignments) and the specific learning domains impacted remain unclear. This, coupled with the potential impact that various treatment regimens can have on learning, suggests that objective assessments of attention and anxiety and broad cognitive domains may be warranted in this group. Similarly, it is unclear if parental reporting of mood concerns within their children is potentially a result of their child’s frustration with schoolwork, disease-specific worries, the parents’ own stress, or a combination of factors. 

As anticipated, pain emerged as a frequent area of difficulty for both adult and pediatric patients, with many reporting that pain interfered with their mood and daily lives. Currently, there is no cure for locally advanced or metastatic MTC; thus, patients must manage living with this disease, often for many years. This concept of balancing medical and social-emotional needs with the desire to maintain a sense of normalcy was echoed by patients and parents in their responses to qualitative probes. Particularly revealing were the ways the participants described the burden of multiple hospital visits, uncertainty regarding their futures, and the impact of physical symptoms. In this context, increased support (e.g., pain management, access to therapeutic services) is an important consideration for practitioners across disciplines and their efforts to improve patient functionality and overall quality of life. A critical element to providing this increased support is accurate and consistent measurement of symptoms, quality of life, and psychological distress over time through patient- and observer-reported outcome measures [40,41]. Our sample’s qualitative responses are consistent with the current literature surrounding MEN2 patients’ reports of psychological distress related to genetic testing and treatment. Specifically, MEN2 patients have reported that initial diagnosis-related stressors lessen over time; however, fear of recurrence and guilt of transmission to children appears to persist [32]. 

We acknowledge several limitations present in this study. First, there is the potential that this sample population is biased towards patients who felt well enough or had the capacity and means to visit NIH and participate in this study; we may not be fully capturing the range of disease progression and status in this population. Similarly, while this study provides valuable insights into patients’ experiences at a single point in time, there is a need for prospective, longitudinal studies to show how psychosocial strengths and vulnerabilities may change over time [42]. Next, our patient sample was not comprised of a diverse racial and ethnic population, which could influence the generalizability of these results. For future studies, we also recognize the need to collect and evaluate relevant disease and treatment variables. This should include the number of surgeries, chemotherapy, and targeted therapies which may be impactful to cognitive, functional, and social-emotional factors and in determining which symptoms could be biological in nature. We only collected self-reported data for children over the age of 12. How children feel and function is critical to understanding their experience of the illness, and future studies should attempt to capture the self-reported experiences of younger children. Finally, collaboration across treatment centers and harmonization of measures used to assess psychosocial and cognitive impacts are also important next steps.

## 5. Conclusions

MEN2-associated MTC is a rare disease that presents with a number of physical symptoms including changes in physical attributes, limitations, and pain. The present study clearly indicates that the psychological impact of living with MEN2 and MTC extends far beyond these areas. Patients with MTC must balance the burden of their medical and educational needs, unique psychosocial concerns, and uncertainty of the future living with a rare and hereditary syndrome. The challenges described by patients in this study are opportunities for clinical providers. Ongoing, patient-centered education about MTC and symptom management, access to mental health resources, and continued research are paramount in the continued improvement of quality of life for those living with MTC. 

## Figures and Tables

**Table 1:** Demographic characteristics of pediatric and adult patients with Medullary Thyroid Carcinoma (MTC).

	Total Sample		Pediatric (<12 Years)		Pediatric (12–17 Years)		Adult Sample (≥18 Years)	
Characteristic	*n*	%	*n*	%	*n*	%	*n*	%
Total N	63	100	15	23.8	31	49.2	17	26.9
Diagnosis MEN2B MEN2A								
	49	77.8	10	66.7	27	87.1	12	70.6
	14	22.2	5	33.3	4	12.9	5	29.4
MTC Diagnosis Yes No								
	52	82.5	10	66.7	28	90.3	14	82.4
	11	17.5	5	33.3	3	9.7	3	17.6
Age								
Mean (M)	16.4		8.5		14.9		26.0	
Standard Deviation (SD)	8.2		2.4		1.4		9.5	
Range	2.7–49.1		2.7–11.9		12.1–17.9		18.2–49.1	
Gender								
*n*	63		15		31		17	
% Male	31	49.2	8	53.3	14	45.2	9	52.9
% Female	32	50.8	7	46.7	17	54.8	8	47.1
Race								
*n*	63		15		31		17	
% White	45	71.4	12	80.0	22	71.0	11	64.7
% Black/African American	5	7.9	0	0	3	9.7	2	11.8
% Latino/a	8	12.7	1	6.7	4	12.9	3	17.6
% Asian/Pacific Islander	3	4.8	2	13.3	0	0	1	5.9
% Other	2	3.2	0	0	2	6.5	0	0
Received Special Education								
*n*	59		14		30		15	
% Yes	15	25.4	4	28.6	7	23.3	4	26.7
% No	44	74.6	10	71.4	23	76.7	11	73.3
Highest Education Completed *								
*n*							14	
% Less than high school							1	7.1
% Graduated high school/GED **							4	28.6
% Some college/vocational							3	21.4
% Graduated college/vocational							4	28.6
% Some professional/graduate							0	0
% Graduate/professional degree							2	14.3

* Adult participants only. ** General Educational Development.

**Table 2:** Adult self-report of psychosocial symptoms.

Symptom	Adult Self Report ^1^ ≥18 Years (*n* = 15)	
	*n*	%
Mood shifts	3	20.0
Attention difficulties	3	20.0
Cries or upset easily	6	40.0
Difficulty concentrating	5	33.3
Anxiety or panic attacks	4	26.7
Sad/Depressed	5	33.3
Loss of interest or pleasure in activities	2	13.3
Feeling hopeless	1	6.7
Difficulty making friends	1	6.7
Difficulty keeping friends	3	20.0

^1^ Valid percentage reported.

**Table 3:** Mental health treatment characteristics.

	Pediatric (*n* = 46) ^1^		Adult (*n* = 17)	
	*n*	% ^2^	*n*	%
Receiving mental health treatment	10	21.7	3	17.6
Taking medication for anxiety	5	10.9	2	11.8
Taking medication for depression	3	6.5	3	17.6
Taking medication for attention difficulties	2	4.3	2	11.8

^1^ Based on parent/caregiver responses for pediatric patients < 18 years. ^2^ Percentage of the total pediatric sample (*n* = 46).

**Table 4:** Pediatric and parent report of psychosocial symptoms.

Symptom	Pediatric Self Report ^1^ 12–17 Years (*n* = 26)		Parents of ^1^ Child 12–17 (*n* = 30)		Parents of Child <12 Years (*n* = 15)	
	***n***	**%**	***n***	**%**	***n***	**%**
Mood shifts	7	26.9	17	56.7	7	46.7
Attention difficulties	13	50.0	9	30.0	3	20.0
Cries or upset easily	10	38.5	12	40.0	6	40.0
Difficulty concentrating	17	65.4	10	33.3	3	20.0
Anxiety or panic attacks	8	30.8	8	26.7	3	20.0
Sad/Depressed	8	30.8	6	20.0	5	33.3
Loss of interest or pleasure in activities	7	26.9	4	13.3	0	0
Feeling hopeless	2	7.7	4	13.3	3	20.0
Difficulty making friends	3	11.5	8	26.7	5	33.3
Difficulty keeping friends	5	19.2	6	20.0	4	26.7

^1^ Valid percentage reported.

**Table 5:** Patient and parent samples of thematic codes.

Theme	Code	Sample
Disease related experiences and challenges	Diagnosis specific concerns	“general sense of illness” (parent) “he will never be cured” (parent) “stress of knowing I have a rare disease” (patient 12–17)
	Treatment Impact	“needles, iv, doctors’ visits all the time” (parent) “keeping my medicine organized” (patient 18+) “all the tests, especially needles” (patient 12–17)
	Symptom Impact	“the constant pain” (parent) “physical issues of colon/urinary problems” (parent) “sleep (not sleeping well)” (patient 12–17)
	Physical Limitations	“unable to do things—physical weakness” (parent) “not able to follow the rhythm of peers of my age (physical activity)” (patient 18+) “Not as physically strong as I’d like to be” (patient 12–17)
Internalized experiences of living with MTC (e.g., sadness, depression)	Mental Health Impact	“sadness” (parent) “social/emotional wellbeing” (parent) “overthinking, stress” (patient 18+) “constant worries” (patient 12–17)
	Coping with Uncertainty	“wondering what’s going to happen” (parent) “not knowing what will happen from scan to scan” (parent) “doubt with testing and what comes next” (patient 18+) “don’t know what the future will bring” (patient 12–17)
	Being and feeling different	inside his mouth that its visible, teeth difference” (parent) “noticing that he is different (physically)” (parent) “people looking and staring” (patient 12–17)
External experiences and challenges of living with MTC (e.g., relationships and school/work environment)	Social Impact	“he has to deal with bedwetting with his friends” (parent) “embarrassment of gas” (parent) “sacrificing a lot of time with friends and family” (patient 18+) “I hate people feeling sorry for me” (patient 12–17)
	Family Impact	“worries about how it affects parents” (parent) “feel guilty for having more attention than my brothers- take up my parents time” (patient 18+) “stress on my family (siblings)” (patient 12–17)
	School/Work-Related Impact	“ struggling with ADHD * and school” (parent) “maintaining school alongside doctor visits” (patient 18+) “miss school- make up work, if I miss too many classes I have to repeat 9th grade” (patient 12–17)

* Attention-deficit/hyperactivity disorder.

## Data Availability

Data from this study will be provided, by the authors, by request.
